# An Inverse Agonist GSK5182 Increases Protein Stability of the Orphan Nuclear Receptor ERRγ via Inhibition of Ubiquitination

**DOI:** 10.3390/ijms24010096

**Published:** 2022-12-21

**Authors:** Soon-Young Na, Ki-Sun Kim, Yoon Seok Jung, Don-Kyu Kim, Jina Kim, Sung Jin Cho, In-Kyu Lee, Jongkyeong Chung, Jeong-Sun Kim, Hueng-Sik Choi

**Affiliations:** 1 School of Biological Sciences and Technology, Chonnam National University, Gwangju 61186, Republic of Korea; 2 Department of Integrative Food, Bioscience and Biotechnology, Chonnam National University, Gwangju 61186, Republic of Korea; 3 New Drug Development Center, Daegu Gyeongbuk Medical Innovation Foundation, Daegu 41061, Republic of Korea; 4 Center for Brain Disorders, Brain Science Institute, Korea Institute of Science and Technology, Seoul 02792, Republic of Korea; 5 Department of Internal Medicine, School of Medicine, Kyungpook National University, Kyungpook National University Hospital, Daegu 41944, Republic of Korea; 6 Research Institute of Aging and Metabolism, Kyungpook National University, Daegu 41940, Republic of Korea; 7 SRC Center for Systems Geroscience, Institute of Molecular Biology and Genetics, School of Biological Sciences, Seoul National University, Seoul 08826, Republic of Korea; 8 Department of Chemistry, Chonnam National University, Gwangju 61186, Republic of Korea

**Keywords:** nuclear receptor, ERRγ, inverse agonist, protein stability, Parkin, ubiquitination

## Abstract

The orphan nuclear receptor, estrogen-related receptor γ (ERRγ) is a constitutively active transcription factor involved in mitochondrial metabolism and energy homeostasis. GSK5182, a specific inverse agonist of ERRγ that inhibits transcriptional activity, induces a conformational change in ERRγ, resulting in a loss of coactivator binding. However, the molecular mechanism underlying the stabilization of the ERRγ protein by its inverse agonist remains largely unknown. In this study, we found that GSK5182 inhibited ubiquitination of ERRγ, thereby stabilizing the ERRγ protein, using cell-based assays and confocal image analysis. Y326 of ERRγ was essential for stabilization by GSK5182, as ligand-induced stabilization of ERRγ was not observed with the ERRγ-Y326A mutant. GSK5182 suppressed ubiquitination of ERRγ by the E3 ligase Parkin and subsequent degradation. The inhibitory activity of GSK5182 was strong even when the ERRγ protein level was elevated, as ERRγ bound to GSK5182 recruited a corepressor, small heterodimer partner-interacting leucine zipper (SMILE), through the activation function 2 (AF-2) domain, without alteration of the nuclear localization or DNA-binding ability of ERRγ. In addition, the AF-2 domain of ERRγ was critical for the regulation of protein stability. Mutants in the AF-2 domain were present at higher levels than the wild type in the absence of GSK5182. Furthermore, the ERRγ-L449A/L451A mutant was no longer susceptible to GSK5182. Thus, the AF-2 domain of ERRγ is responsible for the regulation of transcriptional activity and protein stability by GSK5182. These findings suggest that GSK5182 regulates ERRγ by a unique molecular mechanism, increasing the inactive form of ERRγ via inhibition of ubiquitination.

## 1. Introduction

The estrogen-related receptors (ERRs—ERRα, NR3B1; ERRβ, NR3B2; and ERRγ, NR3B3) are a subfamily of orphan nuclear receptors (NRs) for which the endogenous ligands have not been identified. ERRs are enriched in tissues with high metabolic demands, such as heart, kidney, and skeletal muscle tissues [1,2]. Recent works have shown that ERRγ is a key regulator of diverse metabolic pathways [3]. Furthermore, ERRγ contributes to pathological conditions, such as insulin resistance, alcoholic liver injury, and bacterial infection, suggesting that it represents a possible therapeutic target [4,5,6,7]. ERRγ, like ERRα and ERRβ, constitutively activates transcription in the absence of any ligands. The crystal structure of the ERRγ ligand-binding domain (LBD) reveals that the orientation of the conserved AF-2 domain allows the recruitment of coactivators [8]. No natural ligands of ERRγ have been identified to date, but several small molecules are known to activate or repress the activity of ERRγ. 4-hydroxytamoxifen (4-OHT), originally identified as a partial agonist or antagonist of estrogen receptor alpha (ERα) depending on the tissue, acts as an inverse agonist of ERRγ [9]. GSK5182, one of the 4-OHT derivatives designed to develop selective ERRγ inverse agonists, has a 25-fold higher binding affinity for ERRγ than for ERα [10]. Relative to unliganded or agonist-bound ERRγ, GSK5182 and 4-OHT rearrange the AF-2 domain, resulting in a loss of coactivator binding [11].

Peroxisome proliferator-activated receptor gamma coactivator-1 alpha (PGC-1α) is a transcriptional coactivator that plays a key role in energy homeostasis by regulating fat and glucose metabolism. PGC-1α was found to be strongly induced in the livers of fasting mice, and this led to the expression of gluconeogenic enzymes, such as phosphoenolpyruvate carboxykinase (PEPCK) and glucose-6-phosphatase (G6Pase), to promote hepatic glucose production [12]. Small heterodimer partner-interacting leucine zipper protein (SMILE/CREBZF/Zhangfei) belongs to the CREB/ATF family of basic region leucine zipper (bZIP) transcription factors [13]. SMILE associates with NRs, including ERRγ, and inhibits NR-mediated transactivation by acting as a corepressor [14]. Moreover, SMILE is induced in the liver by feeding and insulin, and overexpression of SMILE decreases hepatic gluconeogenic gene expression by suppressing hepatocyte nuclear factor-4 (HNF-4) transcriptional activity via competition with PGC-1α [15]. Therefore, SMILE counteracts the effect of PGC-1α by balancing hepatic glucose production under different nutritional statuses. Parkin is an E3 ubiquitin ligase mutations to which cause Parkinson’s disease (PD). In SH-SY5Y cells, Parkin controls ROS levels and oxidative stress, suppressing dopamine toxicity by decreasing the expression of monoamine oxidase (MAO), which is responsible for the oxidative deamination of dopamine [16]. All three ERRs significantly induce two MAO isoforms, MAO-A and -B. Parkin directly interacts with ERRs and promotes their ubiquitination and degradation, thereby limiting the expression of MAO-A and -B [17].

In this study, we examined the ligand-dependent turnover of ERRγ protein and found that treatment with GSK5182, a specific inverse agonist of ERRγ, increased the protein level of ERRγ by suppressing its ubiquitination. The inhibitory capacity of GSK5182 was strong even when the protein level of ERRγ was elevated, as GSK5182-bound ERRγ preferred to recruit the corepressor SMILE rather than the coactivator PGC-1α. GSK5182 blocked ERRγ ubiquitination by inhibiting its association with Parkin. Our findings suggest the molecular mechanism by which GSK5182 inhibits ERRγ via increasing the level of the inactive form of ERRγ and preventing its degradation.

## 2. Results

### 2.1. An Inverse Agonist, GSK5182, Stabilizes the ERRγ Protein

**Figure 1:**
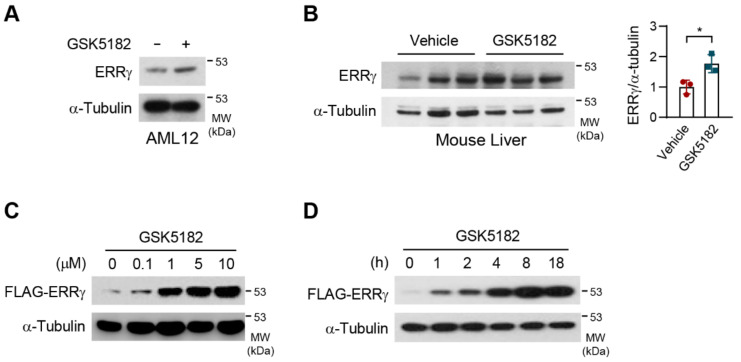
An inverse agonist, GSK5182, increases estrogen-related receptor γ (ERRγ) protein levels. (**A**) AML12 cells were treated with GSK5182 (10 µM) for 24 h and then were analyzed by immunoblotting. (**B**) Vehicle or GSK5182 was injected intraperitoneally at 40 mg/kg/day for 4 days in mice (*n* = 3). Liver lysates were subjected to immunoblotting. Quantification of ERRγ expression normalized against α-tubulin was shown in the right panel. (**C**,**D**) 293T cells transfected with FLAG-ERRγ were treated with the indicated concentration of GSK5182 for 18 h and with GSK5182 (10 µM) for the indicated time, and then were analyzed by immunoblotting. Error bars represent means ± SDs. * *p* < 0.05 by two-tailed unpaired Student’s *t*-test. Data are representative of three independent experiments.

To understand the inhibitory mechanism of an inverse agonist, GSK5182, we investigated whether this compound would regulate the protein stability of ERRγ. Surprisingly, GSK5182 increased the level of ERRγ protein in AML12 mouse hepatocyte cells (Figure 1A). Moreover, when GSK5182 was administered intraperitoneally to mice once daily for 4 days, it increased the level of endogenous ERRγ protein in the liver (Figure 1B). In ERRγ-overexpressed 293T cells, GSK5182 robustly induced the protein in a dose- and time-dependent manner (Figure 1C,D). A substantial increase in ERRγ protein level was seen after 1 h of treatment with 10 µM of GSK5182 (Figure 1D). These results consistently indicate that the inverse agonist GSK5182 increases endogenous and exogenous ERRγ protein levels in liver tissue and cells and suggest that this effect may be a consequence of protein stabilization.

### 2.2. Different Effect of Ligands on ERRγ Protein

**Figure 2:**
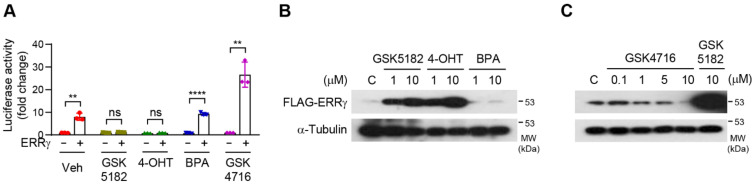
Effects of diverse ligands on ERRγ protein. (**A**) 293T cells were transfected with an expression vector for the Gal4-ERRγ and Gal4-Luc reporter. Luciferase assay was performed following treatment with vehicle, GSK5182, 4-OHT (4-hydroxytamoxifen), BPA (bisphenol A), or GSK4716 (each, 10 µM) for 18 h. (**B**) 293T cells transfected with FLAG-ERRγ were treated with the indicated concentrations of GSK5182, 4-OHT, and BPA for 18 h and then were analyzed by immunoblotting. (**C**) 293T cells transfected with FLAG-ERRγ were treated with the indicated concentrations of GSK4716 and GSK5182 for 18 h and then were analyzed by immunoblotting. Error bars represent means ± SDs. ** *p* < 0.01 and **** *p* < 0.0001 by two-tailed unpaired Student’s *t*-tests; ns, not significant. Experiments were performed in triplicate. Data are representative of three independent experiments.

Although various small molecules have been reported to regulate the transcriptional activity of ERRγ [18], no study to date has directly compared the influence of ligands on the stability of ERRγ. Hence, we investigated whether various ligands could impact the protein stability of ERRγ. First, we confirmed that ERRγ activity was differentially regulated by treatment with GSK5182, 4-OHT, BPA, and GSK4716. As expected, GSK5182 and 4-OHT significantly inhibited the transcriptional activity of ERRγ, whereas the known agonist GSK4716 increased it. Consistent with a previous report [19], BPA did not affect the transcriptional activity of ERRγ (Figure 2A). We then evaluated the effect of 4-OHT, BPA, and GSK4716 on ERRγ protein. 4-OHT augmented ERRγ protein levels relative to GSK5182, but BPA had no effect (Figure 2B). By contrast, GSK4716 gradually induced the degradation of ERRγ in a dose-dependent manner (Figure 2C). Taken together, these results demonstrate that ERRγ ligands exert various effects on protein stability and, in particular, reveal an inverse relationship between transcriptional activity and receptor stability.

### 2.3. Protein Stability Due to GSK5182 Requires Y326 of ERRγ

**Figure 3:**
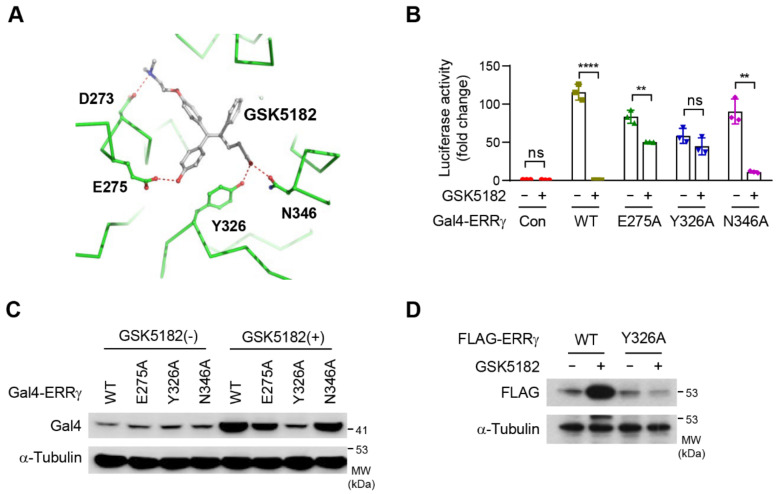
Upregulation of ERRγ protein levels by GSK5182 requires Y326. (**A**) ERRγ LBD (green) is shown in a ribbon diagram with GSK5182 (gray carbons), and the residues involved in polar interactions (green carbons) are displayed as ball-and-stick models. Hydrogen bonds between molecules are shown as red dotted lines. This figure was prepared using the PyMol molecular graphics program (Schrödinger, LLC). (**B**) 293T cells were transfected with expression vectors for Gal4-ERRγ-WT (wild type) or Gal4-ERRγ-mutants and the Gal4-Luc reporter. Luciferase reporter assays were performed following treatment with vehicle or GSK5182 (1 µM) for 18 h. (**C**) 293T cells transfected with Gal4-ERRγ-WT or Gal4-ERRγ-mutants were treated with vehicle or GSK5182 (1 µM) for 18 h and then were analyzed by immunoblotting. (**D**) 293T cells transfected with FLAG-ERRγ-WT or FLAG-ERRγ-Y326A were treated with vehicle or GSK5182 (1 µM) for 18 h and then were analyzed by immunoblotting. Error bars represent means ± SDs. ** *p* < 0.01 and **** *p* < 0.0001 by two-tailed unpaired Student’s *t*-tests; ns, not significant. Experiments were performed in triplicate. Data are representative of three independent experiments.

As an inverse agonist, GSK5182 is selective for ERRγ relative to ERα due to its additional hydrogen-bonding interactions with Y326 and N346 (Figure 3A) [10]. Previously, we demonstrated that the inhibitory effect of GSK5182 depended on the interaction with Y326 of ERRγ [20]. To determine whether the residues involved in the ERRγ-GSK5182 interaction were also required for protein stabilization, we introduced mutations that disrupted the GSK5182 contact sites. The transcriptional activity of the mutated receptors was tested by a reporter assay alongside wild-type (WT) ERRγ (Figure 3B). E275A and N346A had approximately 80% activity, and Y326A had 50% activity relative to the wild type. GSK5182 strongly inhibited wild-type Gal4-ERRγ and N436A and partially inhibited E275A, but did not inhibit the activity of Y326A, confirming that Y326 is a critical residue for GSK5182-mediated inhibition (Figure 3B). We then subjected the mutants to Western blotting to investigate the change in protein stability induced by GSK5182. The mutants were expressed at similar levels (Figure 3C, lanes 1–4), and GSK5182 increased the levels of the wild type, E275A, and N346A, but not Y326A, indicating that mutation of Y326 abolished ligand-induced stabilization (Figure 3C). This finding was also confirmed with FLAG-ERRγ-Y326A (Figure 3D). Interestingly, the increase in the protein levels of wild-type and mutant ERRγ by GSK5182 was inversely proportional to the degree of transcriptional inhibition. Thus, Y326 is important not only for GSK5182-mediated inhibition of ERRγ but also for GSK5182-induced stabilization of the protein.

### 2.4. GSK5182 Stabilizes ERRγ by Extending Its Half-Life

**Figure 4:**
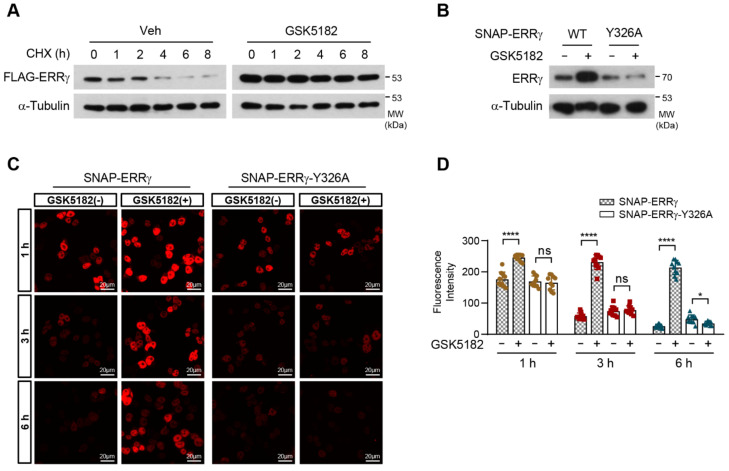
GSK5182 increases the protein stability of ERRγ. (**A**) 293T cells transfected with FLAG-ERRγ were incubated overnight with vehicle or GSK5182 and then were treated with cycloheximide (CHX, 10 µg/mL) for the indicated times in the absence or presence of GSK5182 (10 µM). Cell lysates were subjected to immunoblotting. (**B**) 293T cells transfected with the pSNAP-ERRγ-WT or pSNAP-ERRγ-Y326A were treated with vehicle or GSK5182 (1 µM) for 10 h and then were analyzed by immunoblotting. (**C**) 293T cells transfected with SNAP-ERRγ and SNAP-ERRγ-Y326A were incubated with vehicle or GSK5182 (1 µM) for 10 h. Cells were then labeled with SNAP-Cell TMR-Star for 10 min, washed, and incubated for the indicated times in the absence of GSK5182. The samples were fixed with 4% formaldehyde and subjected to confocal microscopy analysis. (**D**) To analyze the fluorescence intensity of transfected cells at each time point, the mean values of fluorescence intensity were analyzed in 10 confocal images in which 10 elliptical ROIs (regions of interest) of 10.47 µm^2^ were drawn within the nuclear regions of transfected cells in each image. The mean values were measured using the Quantify module of the LAS AF program. Error bars represent means ± SDs. * *p* < 0.05 and **** *p* < 0.0001 by two-tailed unpaired Student’s *t*-tests; ns, not significant. Data are representative of three independent experiments.

To determine the effect of the ligand on receptor stability, we monitored ERRγ protein stability by treating cells with the protein synthesis inhibitor cycloheximide (CHX) in the absence or presence of GSK5182. ERRγ protein levels were greatly reduced within 4 h of CHX treatment (Figure 4A, lanes 1–6). Incubation with GSK5182 increased ERRγ protein levels and prevented CHX-induced decline beyond 8 h (Figure 4A, lanes 7–12). Thus, GSK5182 increased the receptor protein level by extending its half-life. We then performed pulse labeling with the SNAP-tag and followed the decay of the labeled protein to measure the rate of degradation of SNAP-ERRγ in 293T cells. We first confirmed that GSK5182 increased the level of SNAP-ERRγ protein, whereas the level of SNAP-ERRγ-Y326A protein, in which the GSK5182 contact site was disrupted, remained unchanged in the presence of GSK5182, consistent with the results for FLAG-ERRγ-Y326A shown in Figure 3D (Figure 4B). Similar to the Western blotting results in Figure 4A, TMR-Star-labeled SNAP-ERRγ was diminished by over 50% after 3 h of incubation. By contrast, SNAP-ERRγ in the presence of GSK5182 remained almost unchanged over 6 h. GSK5182 did not affect the protein decay of SNAP-ERRγ-Y326 (Figure 4C,D). These results suggest that GSK5182 stabilizes ERRγ by preventing its degradation and extending the protein’s half-life.

### 2.5. GSK5182 Prevents ERRγ Ubiquitination by Inhibiting Its Association with the E3 Ligase Parkin

**Figure 5:**
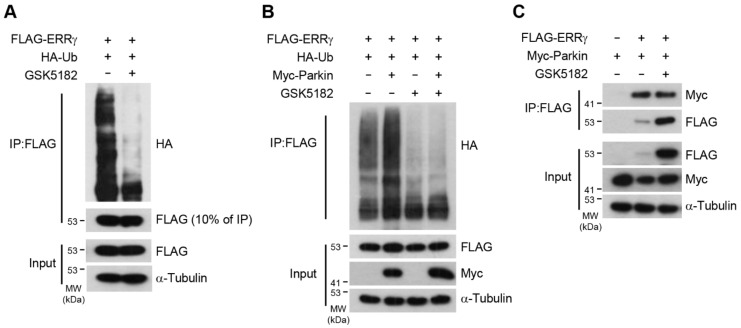
GSK5182 prevents ERRγ degradation, blocking its ubiquitination by inhibiting association with the E3 ligase Parkin. (**A**) 293T cells transfected with expression vectors for FLAG-ERRγ and HA-ubiquitin were treated with vehicle or GSK5182 (10 µM) for 18 h. Cell lysates were immunoprecipitated with anti-FLAG and then were analyzed by immunoblotting with anti-HA. (**B**) 293T cells transfected with FLAG-ERRγ, HA-Ub, and Myc-Parkin were treated with vehicle or GSK5182 (10 µM) for 18 h. Cell lysates were immunoprecipitated with anti-FLAG and then were analyzed by immunoblotting with anti-HA. (**C**) 293T cells were transfected with expression vectors for FLAG-ERRγ and Myc-Parkin and were incubated with vehicle or GSK5182 (10 µM) for 18 h. Cell lysates were immunoprecipitated with anti-FLAG and then analyzed by immunoblotting. Data are representative of three independent experiments.

Proteasomal degradation requires the covalent attachment of ubiquitin (Ub) chains. Given that GSK5182 promoted the accumulation of ERRγ protein, we next asked whether GSK5182 would prevent the ubiquitination of ERRγ. Since GSK5182 strongly increased the protein level, to compare ubiquitination between ERRγ and ERRγ with GSK5182, we adjusted the amount of transfected DNA to achieve equal expression levels (Figure 5A). GSK5182 treatment blocked ubiquitination of ERRγ, suggesting that GSK5182 increased the level of ERRγ protein by preventing ubiquitin-mediated proteasome degradation (Figure 5A). A previous study reported that Parkin E3 ligase directly ubiquitinated and degraded ERRα and ERRγ, resulting in suppression of monoamine oxidases (MAOs) [17]. Hence, we investigated whether GSK5182 interrupted Parkin-mediated ubiquitination of ERRγ. Parkin increased ubiquitination of ERRγ, but its effect was abolished in the presence of GSK5182 (Figure 5B). Furthermore, the direct interaction between ERRγ and Parkin was reduced by GSK5182 treatment (Figure 5C). These results indicate that GSK5182 prevents ubiquitination of ERRγ by inhibiting its association with Parkin.

### 2.6. GSK5182 Promotes Recruitment of the Corepressor SMILE to ERRγ

**Figure 6:**
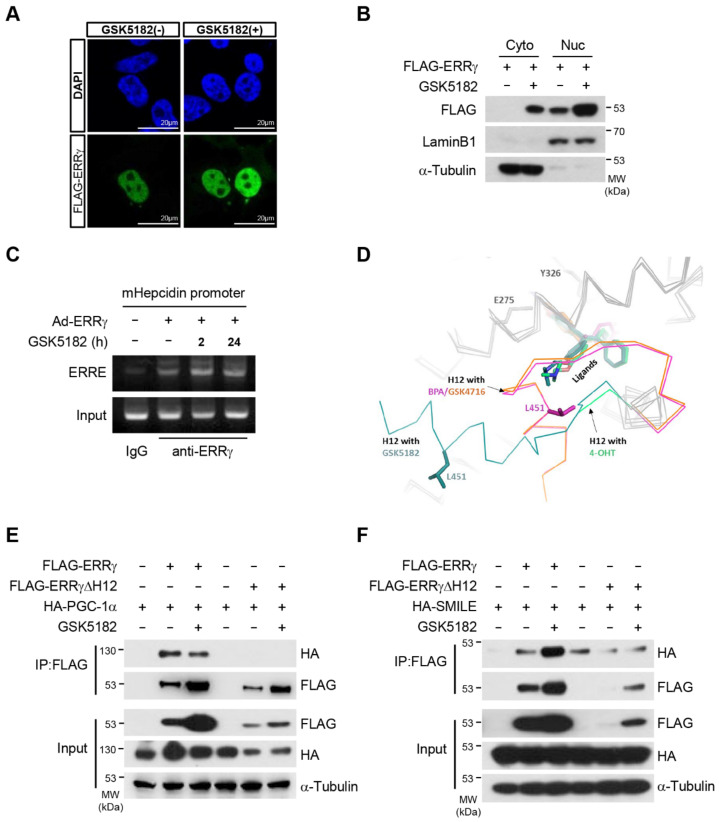
GSK5182 does not hinder subcellular localization and DNA binding of ERRγ but promotes the binding of the corepressor to ERRγ. (**A**) Nuclear localization of ERRγ in the absence or presence of GSK5182. 293T cells transfected with FLAG-ERRγ were treated with vehicle or GSK5182 (10 µM) for 18 h and then were subjected to confocal microscopy. Cells were stained with Alexa Fluor 488-conjugated anti-FLAG (bottom panels; green), and nuclear DNA was stained with DAPI (top panels; blue). (**B**) 293T cells transfected with FLAG-ERRγ were treated with vehicle or GSK5182 (10 µM) for 18 h. Nuclear or cytoplasmic fractions were analyzed by immunoblotting. Lamin B1 and α-Tubulin were used as nuclear and cytosolic markers, respectively. (**C**) AML12 cells infected with Ad-ERRγ were treated with vehicle or GSK5182 (10 µM) for the indicated times. A ChIP assay was performed to detect the binding of ERRγ to the mouse *hepcidin* promoter. Soluble chromatin was subjected to immunoprecipitation using anti-IgG or anti-ERRγ. After DNA recovery, DNA was amplified by PCR using primers of the *hepcidin* promoter and analyzed by agarose gel electrophoresis. (**D**) ERRγ LBD in complex with diverse ligands. The ligand-bound structures of ERRγ LBDs were superposed using PyMol (Schrödinger, LLC). The LBDs were drawn as ribbons and some key residues were drawn as stick models. The H12 helices are highlighted and labeled by alternating colors based on the bound ligands: 4-OHT (lime, PDB ID 2p7z), GSK5182 (teal, PDB ID 2ewp), BPA (magenta, PDB ID 2p7g), and GSK4716 (orange, PDB ID 2gpp). (**E**) 293T cells transfected with FLAG-ERRγ or FLAG-ERRγΔH12with HA-PGC-1α were treated with vehicle or GSK5182 (10 µM) for 18 h. Cell lysates were immunoprecipitated with anti-FLAG and then were analyzed by immunoblotting with anti-HA and anti-FLAG. (**F**) 293T cells transfected with FLAG-ERRγ or FLAG-ERRγΔH12 with HA-SMILE were treated with vehicle or GSK5182 (10 µM) for 18 h. Cell lysates were immunoprecipitated with anti-FLAG and then were analyzed by immunoblotting with anti-HA and anti-FLAG. Data are representative of three independent experiments.

Given that GSK5182 strongly inhibited ERRγ transcriptional activity while increasing the level of ERRγ protein, we investigated whether cellular localization and the DNA-binding ability of ERRγ were altered in the presence of GSK5182. Immunostaining assays confirmed that ERRγ localized uniformly in the nucleus in the absence or presence of GSK5182 (Figure 6A). Fractionation of nuclei and cytoplasm revealed that the receptor was partly localized in the cytoplasm but that mostly it was in the nucleus (Figure 6B). Next, we performed ChIP experiments to determine whether GSK5182 could affect the ability of ERRγ to bind its target promoters. As shown in Figure 6C, ERRγ occupied the mouse *hepcidin* promoter [5], and neither short- nor long-term incubation with GSK5182 affected this binding. These data suggested that GSK5182 did not change the subcellular localization or DNA-binding ability of ERRγ. 

Structural studies have shown that unliganded ERRγ LBD has a conformation similar to those of agonist-bound NRs, allowing the coactivator to bind to the pocket in the LBD [11]. In crystal structures of ERRγ LBD in complex with 4-OHT, GSK5182, BPA, and GSK4716, the spatial positions of helix 12 (H12) varied depending on the bound ligand (Figure 6D). Binding of the agonist GSK4716 [11] or BPA [21] to the ERRγ LBD did not rearrange the C-terminal H12, consistent with the configuration of unliganded ERRγ. By contrast, binding of the inverse agonist GSK5182 [10] or 4-OHT [21] to the ERRγ LBD induced local rearrangement of H12, yielding a conformation that did not favor the binding of a coactivator. As the AF-2 domain in H12 is responsible for the recruitment of coactivators or corepressors, we investigated whether GSK5182 influenced cofactor binding to ERRγ. GSK5182 treatment disrupted the interaction between ERRγ and PGC-1α, a coactivator, despite high levels of ERRγ protein (Figure 6E,F). By contrast, the interaction of ERRγ with SMILE, a corepressor, was strengthened as the level of ERRγ protein increased in response to GSK5182. Deletion of H12 abolished the interaction with both PGC-1α and SMILE. This suggested that the changed conformation of ERRγ by GSK5182 preferred binding to the corepressor instead of the coactivator. Taken together, these results demonstrate that GSK5182 exerts its strong inhibitory action by recruiting the corepressor to ERRγ, even when the protein level is elevated.

### 2.7. The AF-2 Domain of ERRγ Is Crucial for Protein Turnover

**Figure 7:**
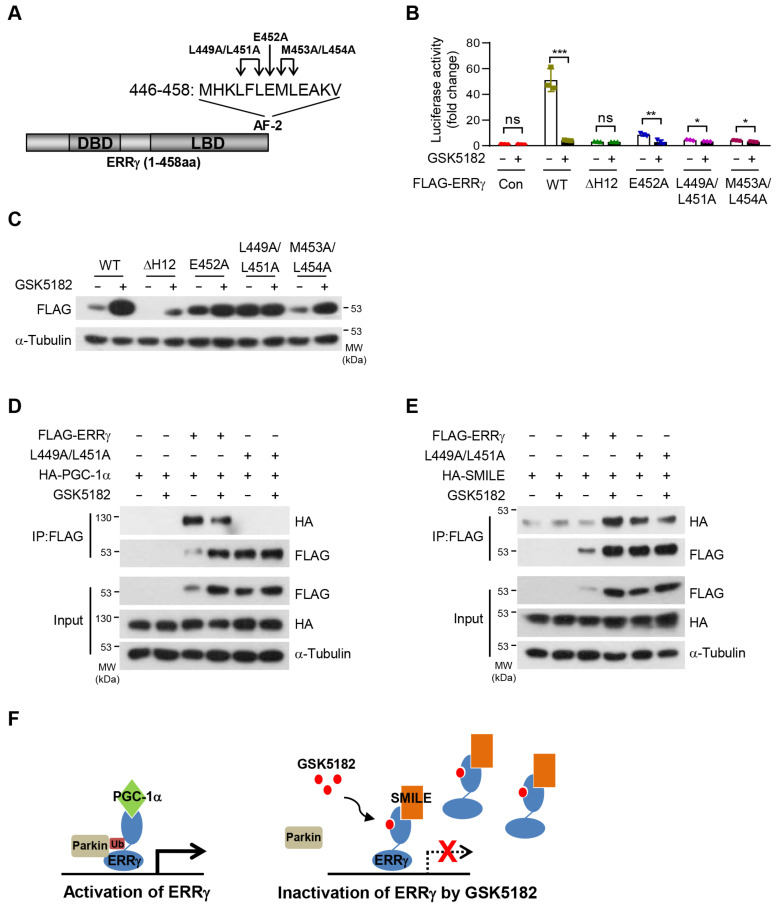
The AF-2 domain of ERRγ regulates the effect of GSK5182 on the protein stability of ERRγ dependent on corepressor recruitment. (**A**) Schematic representation of the structure of ERRγ and the AF-2 domain. (**B**) 293T cells were transfected with expression vectors for FLAG-ERRγ or its mutants, along with the sft4-Luc reporter. Luciferase assay was performed following treatment with vehicle or GSK5182 (10 µM) for 18 h. (**C**) 293T cells transfected with FLAG-ERRγ or mutants were treated with vehicle or GSK5182 (10 µM) for 18 h and then were analyzed by immunoblotting. (**D**) 293T cells transfected with FLAG-ERRγ or FLAG-ERRγ-L449A/L451A with HA-PGC-1α were treated with vehicle or GSK5182 (10 µM) for 18 h. Cell lysates were immunoprecipitated with anti-FLAG and then were analyzed by immunoblotting with anti-HA and anti-FLAG. (**E**) 293T cells transfected with FLAG-ERRγ or FLAG-ERRγ-L449A/L451A with HA-SMILE were treated with vehicle or GSK5182 (10 µM) for 18 h. Cell lysates were immunoprecipitated with anti-FLAG and then were analyzed by immunoblotting with anti-HA and anti-FLAG. (**F**) Schematic diagram of the mechanism of ERRγ inhibition by the inverse agonist GSK5182. Error bars represent means ± SDs. * *p* < 0.05, ** *p* < 0.01, and *** *p* < 0.001 by two-tailed unpaired Student’s *t*-tests; ns, not significant. Experiments were performed in triplicate. Data are representative of three independent experiments.

Given that GSK5182 induced a conformational change in ERRγ, specifically the AF-2 domain, we asked whether the AF-2 domain of ERRγ was critical for protein turnover by GSK5182. Deletion and single- or double-amino acid substitution in the AF-2 domain of ERRγ abolished its transcriptional activity (Figure 7A,B). The single mutant E452A and the double mutant L449A/L451A exhibited higher protein levels than the wild type, suggesting that the AF-2 domain of ERRγ was associated with protein turnover (Figure 7C, lanes 5 and 7). The effect of GSK5182 on protein level was significantly diminished with E452A and completely ablated with L449A/L451A (Figure 7C, lanes 6 and 8). Whereas L449A/L451A was unable to interact with PGC-1α (Figure 7D), the interaction between L449A/L451A and SMILE was stronger than for the wild type. However, the L449A/L451A protein level was unchanged by GSK5182, indicating that L449A/L451A was no longer susceptible to GSK5182 (Figure 7E). Taken together, these results demonstrate that the AF-2 domain of ERRγ is responsible for protein turnover and the effect of GSK5182 on the protein level.

## 3. Discussion

In this study, we reveal that, under normal conditions, active ERRγ undergoes constitutive ubiquitination and degradation to maintain appropriate transcriptional activity, interacting with the coactivator PGC-1α. GSK5182 induced a conformational change in ERRγ which inhibited association with the E3 ligase Parkin, thereby blocking ubiquitin-mediated protein degradation and stabilizing inactive form of ERRγ bound to the corepressor SMILE (Figure 7F). Thus, GSK5182 exerts its strong inhibitory effect by increasing the level of inactive form of ERRγ.

Ligands not only control receptor activity but also influence nuclear receptor stability, thus linking transactivation and degradation. NRs are subject to ubiquitin-dependent proteolysis by the 26S proteasome, in both ligand-dependent and -independent manners [22]. Many receptors, including estrogen receptor alpha (ERα), progesterone receptor (PR), glucocorticoid receptor (GR), thyroid receptor (TR), retinoic acid receptor (RAR), and peroxisome proliferator-activated receptor gamma (PPARγ), are downregulated upon cognate ligand binding [23]. Suppression of proteasome-mediated degradation of ligand-bound ERα inhibits transcriptional activity, suggesting that receptor degradation is required for transcriptional activation [24]. On the other hand, a few receptors are upregulated by their ligands. Specifically, short-lived receptors, such as liver X receptor alpha (LXRα) [25] and PPARδ, are stabilized by ligands [26], which causes their transcriptional activity to be sustained for longer. Agonists, antagonists, and selective estrogen receptor modulators (SERMs) of ERα affect stability differently. The agonist E2 promotes degradation of ERα, creating an inverse relationship between transcriptional activity and protein stability. However, this relationship does not apply to antagonists and SERMs, implying that ligand-induced conformational change is a critical determinant of protein stability. Thus, the different conformations of ERα bound to various ligands influence both transcriptional activity and protein stability [27]. ERRγ is an orphan nuclear receptor that has constitutive activity without ligand binding, and its activity is regulated by co-regulators and post-translational modifications (PTMs) [28]. Recent findings suggest that ERRγ is induced in response to nutrient availability, stresses, and metabolic demands. ERRγ upregulates the expression of various target genes involved in pathways such as gluconeogenesis and the metabolism of alcohol, lipids, iron, cholesterol, and so on [3,5,20,29,30,31]. Therefore, it would be valuable to reveal the mechanism of action of GSK5182. Our results show that GSK5182 substantially stabilizes ERRγ, decreasing its ubiquitin-dependent degradation. Moreover, ERRγ bound to GSK5182 prefers to interact with the corepressor SMILE rather than the coactivator PGC-1α. Thus, GSK5182 reinforces its inhibitory activity by stabilizing an inactive form of the receptor.

ERRγ adopts an active conformation in the absence of ligands. The crystal structure reveals that the agonist GSK4716 does not rearrange the AF-2 domain; instead, GSK4176 activates ERRγ through thermal stabilization of the LBD domain [11]. Our results show that GSK4716 promotes protein degradation of ERRγ in a dose-dependent manner (Figure 2C). It is conceivable that GSK4716 facilitates reinitiation of transcription, similar to a mechanism proposed for NRs [32]. In general, nuclear receptor LBDs adopt an antiparallel α-helical sandwich fold consisting of 12 α-helices (H1–H12) and a small β-sheet. Agonists stabilize the AF-2 domain and induces a hydrophobic coactivator binding surface with H12 which is located in the AF-2 domain. While unliganded ERRγ LBD has a conformation similar to agonist-bound NRs, inverse agonist 4-OHT binding to ERRγ induces rotation of the Phe-435 side chain that partially fills the cavity of LBD, resulting in complete dissociation of H12 from the LBD body, so that H12 eventually interferes with coactivator recruitment [33]. Furthermore, the structure in which the corepressor SMRT peptide is added to the ERRγ with 4-OHT complex shows no significant changes in the LBD of ERRγ/4-OHT [11]. The crystal structure of the ERRγ LBD-bound GSK5182 is very similar to the structure of ERRγ/4-OHT; in both, the AF-2 domain is rearranged into the same position as in the unliganded structure of other NRs, enabling recruitment of corepressors (Figure 6D) [10]. Since the changed conformation of ERRγ by GSK5182 prefers binding to the corepressor SMILE instead of the coactivator PGC-1, as shown in Figure 6E,F, these results support the crystal structure, suggesting that the preference for coactivator/corepressor is determined by the location of the AF-2 domain of ERRγ. We observed that GSK5182 did not affect DNA binding or cellular localization (Figure 6A-C) but extended the half-life of ERRγ by blocking ubiquitin-mediated degradation (Figure 5A). Thus, the conformational change in LBD induced by GSK5182 is critical for its activity as an inverse agonist. Our results showing that GSK5182 stabilized ERRγ protein, causing inactive ERRγ to accumulate (Figure 6E,F), have two important implications: (1) that it would be difficult for active ERRγ to access the ERRE of target genes in the presence of a large amount of inactive ERRγ; and (2) that occupation of the ERRE by inactive ERRγ might be prolonged due to the extended protein half-life under inhibitory conditions, during which it is bound to corepressors. Together, these features make GSK5182 a very strong inhibitor of ERRγ. Although ERRγ is ubiquitinated and GSK5182 blocks ubiquitination of ERRγ by inhibiting its association with the E3 ligase Parkin (Figure 5B,C), further investigations are needed to identify the ubiquitination sites to determine whether ubiquitination sites are affected by GSK5182. Recent work showed that newly developed analogs of GSK5182 promote the activity of the sodium iodide symporter (NIS), a key protein in radioiodine therapy in anaplastic thyroid cancer (ATC) cells [34]. In contrast to GSK5182, these analogs promote degradation of the ERRγ protein. Thus, GSK5182 may work via different mechanisms under different physiological conditions.

Ligand binding dramatically displaces the C-terminal AF-2 domain of NRs [35], raising the question of whether the AF-2 domain is responsible for ligand-induced protein degradation or stabilization. The level of PPARγ is significantly reduced in response to thiazolidinediones, and PPARγ degradation correlates with transcriptional activity. This phenomenon is dependent on the AF-2 domain of PPARγ, as demonstrated by the observation that the level of mutant E449Q does not change upon ligand exposure [36]. The AF-2 domain of RARγ2 is also required for both degradation and transactivation [37]. By contrast, the level of the AF-2 mutant L432A/E435A of PPARδ is not affected by a ligand that stabilizes the receptor by blocking ubiquitination [26]. ERα is downregulated in the presence of its cognate ligand, E2. Degradation by the 26S proteasome is necessary for transcriptional activation of ERα. Interestingly, L543A/L544A mutation of ERα in the AF-2 domain changed antagonists, such as fulvestrant/ICI182780 or 4-OHT, into agonists inducing homodimerization in L543A/L544A mutant knock-in mice [38]. Our data suggest that the AF-2 domain of ERRγ is associated with protein degradation (Figure 7C). Levels of the L449A/L451A and E452A mutants were elevated in the absence of GSK5182, implying that the effect of GSK5182 on protein stability was abrogated for both mutants. These observations indicate that the AF-2 domain of ERRγ is involved not only in transcriptional activity but also in protein stability.

Our results suggest a novel mechanism by which an inverse agonist regulates the transcriptional activity and protein stability of ERRγ. As the function of ERRγ is being revealed and its importance is becoming apparent, efforts are being made to develop small molecules that modulate its activity [39,40]. Recently, DN200434, a new derivate of GSK5182 and an orally available inverse agonist of ERRγ, has been proposed as an alternative solution to solve the resistance problem that most liver cancer patients develop tolerance within 6 months of sorafenib administration [40]. Elucidation of the mechanism by which ERRγ is modulated by agonists or inverse agonists will facilitate the development of clinical therapies for ERRγ-related diseases [41,42].

## 4. Materials and Methods

### 4.1. Chemicals

GSK5182 was synthesized at Kyungpook National University (Daegu, Republic of Korea) and DGMIF (Daegu-Gyeongbuk Medical Innovation Foundation, Daegu, Republic of Korea), as described previously [10,34,43,44], and dissolved in 30% polyethylene glycol 400 (PEG400, USB, Cleveland, OH, USA) or DMSO (MilliporeSigma, St. Louis, MO, USA). GSK4716 was purchased from Abcam (Cambridge, UK). 4-hydroxytamoxifen (4-OHT) and bisphenol A (BPA) were purchased from MilliporeSigma (St. Louis, MO, USA). XCT790 was purchased from TOCRIS (Bristol, UK) 

### 4.2. Plasmids, DNA Constructs, and Recombinant Adenoviruses

The reporter plasmid sft4-luc (ERRE-luc) and pcDNA3 vectors expressing FLAG-ERRα and FLAG-ERRγ were described previously [45]. An expression vector for Parkin was kindly provided by Dr. Jongkyeong Chung of Seoul National University (Seoul, Republic of Korea). Constructs encoding mutants of Gal4- and FLAG-ERRγ were generated using a QuikChange Lighting Site-Directed Mutagenesis Kit (#210519, Agilent, Santa Clara, CA, USA). For SNAP-ERRγ and SNAP-ERRγ-Y326A, ERRγ and ERRγ-Y326A, respectively, were PCR-amplified and subcloned into pSNAPf (#E9183, NEB, Ipswich, MA, USA) using the XhoI and NotI restriction sites. Adenoviruses expressing control GFP and FLAG-ERRγ were described previously [20]. All viruses were purified using CsCl_2_.

### 4.3. Cell Culture, Transient Transfection, and Luciferase Assay 

HEK 293T cells were cultured in Dulbecco’s modified Eagle’s medium (DMEM; Welgene, Gyeongsan-si, Republic of Korea) supplemented with 10% fetal bovine serum (FBS; Gibco, Waltham, MA, USA). AML12 cells were cultured in Dulbecco’s modified Eagle’s medium: Nutrient Mixture F-12 (DMEM-F12, Welgene, Gyeongsan-si, Republic of Korea) supplemented with 10% FBS, ITSP solution (insulin–transferrin–selenium–pyruvate supplement; Welgene, Gyeongsan-si, Republic of Korea), and 0.1 µM of dexamethasone. Mouse primary hepatocytes were isolated from C57BL/6 mice by collagenase perfusion and seeded with DMEM containing 10% FBS. Transfection of the reporters and expression vectors used in each experiment into cells was performed using Lipofectamine 2000 reagent, according to the manufacturer’s instructions (Invitrogen, Carlsbad, CA, USA), and cells were treated with 10 μM of GSK5182, 4-OHT, BPA, or GSK4716 for 18 h, unless it is noted otherwise. Luciferase activity was measured the next day and normalized against β-galactosidase activity.

### 4.4. Animal Experiments 

Male 8-week-old C57BL/6J mice (The Jackson Laboratory, Bar Harbor, ME, USA) were maintained under a 12:12 h light/dark cycle and fed ad libitum. Vehicle or GSK5182 was administered by intraperitoneal injection at 40 mg/kg/day for 4 days (*n* = 3). Mice were injected with Rompun (Bayer, Leverkusen, Germany) and Zoletil50 (Virbac, Carros, France) and sacrificed by exsanguination according to a protocol approved by the Chonnam National University Animal Care and Use Committee (no. CNU IACUC-YB-2017-42).

### 4.5. Chromatin Immunoprecipitation (ChIP) Assay 

ChIP was performed using the Chromatin IP kit (#9004, Cell Signaling Technology, Danvers, MA, USA). Soluble chromatin was subjected to immunoprecipitation using anti-IgG or anti-ERRγ (#PP-H6812-00, R&D systems, Minneapolis, MN, USA). After DNA recovery, DNA was analyzed by PCR using primers against the hepcidin promoter (forward: 5′-GAGCCACAGTGTGACATCAC-3′; reverse: 5′-GTCTAGGAGCCAGTCCCAGT-3′).

### 4.6. Pulse Labeling 

To trace protein decay, SNAP-tag expression vectors were transfected and incubated with GSK5182 for 10 h before labeling. After 10 min of pulse labeling with SNAP-Cell TMR-Star (#S9105, NEB, Ipswich, MA, USA), a cell-permeable red fluorescent substrate, cells were washed three times. The cells were then incubated in fresh medium for 1 h, and the medium was replaced one more time to remove unreacted SNAP-tag substrate that had diffused out of the cells. Mean values of fluorescence intensity were analyzed using 10 confocal images marked with 10 elliptical ROIs (regions of interest), 10.47 µm^2^ in area per image at each time point. ROIs were drawn within the nuclear regions of transfected cells.

### 4.7. Confocal Microscopy

Twenty-four hours after transfection, cells were fixed with 4% formaldehyde, immunostained, mounted with ProLong Gold (#P36935, Invitrogen, Carlsbad, CA, USA), and observed by confocal microscopy. Confocal images of the SNAP-tag fusion ERRγ construct labeled with SNAP-Cell TMR-Star (#S9105, NEB, Ipswich, MA, USA) were obtained with a Laser Scanning Confocal Microscope System (TCS SP5 AOBS/Tandem, Leica Microsystems, Wetzlar, Germany, at the Korea Basic Science Institute, Gwangju Center). All confocal images were obtained using a 63X objective (1.40 oil UV) under the same hardware parameter settings (gain, power of the 561 nm laser, and spectral emission in the 570–610 nm range). The fluorescence intensities of confocal images were analyzed using the quantification module of LAS AF (Leica Microsystems, Wetzlar, Germany).

### 4.8. Immunoblotting 

Whole-cell extracts were prepared with lysis buffer (50 mM Tris-pH7.5, 150 mM NaCl, 1% NP-40, and 5 mM EDTA). Equal amounts of total proteins were separated by SDS-PAGE and transferred to PVDF membranes. Membranes were immunoblotted with anti-FLAG, anti-HA, anti-Myc (Cell Signaling Technology, Danvers, MA, USA), anti-GFP, anti-Gal4, anti-Lamin B1 (Santa Cruz Biotechnology, Dallas, TX, USA), anti-α-tubulin (AbFrontier, Seoul, Republic of Korea), and anti-ERRγ (R&D Systems, Minneapolis, MN, USA) antibodies. Densitometry was performed using ImageJ software (NIH, Bethesda, AR, USA).

### 4.9. Nuclear/Cytosol Fractionation

Cells were transfected and incubated for 24 h. Nuclear extracts for Western blotting were obtained using a Nuclear/Cytosol fractionation kit (#K266-25, BioVision, Milpitas, CA, USA).

### 4.10. Statistical Analyses

The significance of differences among the mean values for the groups was evaluated by two-tailed unpaired Student’s *t*-tests (Prism 8, GraphPad Software, San Diego, CA, USA). All values were expressed as means ± SDs.

## Data Availability

Not applicable.
